# The Pathophysiologic Role of Gelsolin in Chronic Kidney Disease: Focus on Podocytes

**DOI:** 10.3390/ijms222413281

**Published:** 2021-12-10

**Authors:** Chia-Jung Yu, Dian W. Damaiyanti, Shian-Jang Yan, Chih-Hsing Wu, Ming-Jer Tang, Dar-Bin Shieh, Peter P. Liu, Ping-Yen Liu

**Affiliations:** 1 Institute of Clinical Medicine, College of Medicine, National Cheng Kung University, Tainan 70457, Taiwan; tina_8678@hotmail.com (C.-J.Y.); damaiyanti@hangtuah.ac.id (D.W.D.); 2 Department of Oral Biology, Faculty of Dentistry, Universitas Hang Tuah, Surabaya 60111, Indonesia; 3 Department of Physiology, College of Medicine, National Cheng Kung University, No. 1, University Road, Tainan 70457, Taiwan; johnyan@mail.ncku.edu.tw (S.-J.Y.); mjtang1@mail.ncku.edu.tw (M.-J.T.); 4 Department of Family Medicine, National Cheng Kung University Hospital, College of Medicine, National Cheng Kung University, Tainan 70457, Taiwan; paulo@mail.ncku.edu.tw; 5 Department of Family Medicine, College of Medicine, National Cheng Kung University, Tainan 70457, Taiwan; 6 Institute of Gerontology, College of Medicine, National Cheng Kung University, Tainan 70457, Taiwan; 7 International Center for Wound Repair and Regeneration, National Cheng Kung University, Tainan 70457, Taiwan; 8 Institute of Oral Medicine, National Cheng Kung University, Tainan 70101, Taiwan; dbshieh@mail.ncku.edu.tw; 9 Institute of Basic Medicine, National Cheng Kung University, Tainan 70101, Taiwan; 10 Center of Applied Nanomedicine, National Cheng Kung University, Tainan 70101, Taiwan; 11 Core Facility Center, National Cheng Kung University, Tainan 701401, Taiwan; 12 Department of Stomatology, National Cheng Kung University Hospital, Tainan 704302, Taiwan; 13 Cardiac Function Laboratory, University of Ottawa Heart Institute, 40 Ruskin St., Ottawa, ON K1Y 4W7, Canada; peter.liu@utoronto.ca; 14 Department of Cellular and Molecular Medicine, University of Ottawa, Ottawa, ON K1Y 4W7, Canada; 15 Division of Cardiology, Internal Medicine, National Cheng Kung University Hospital, College of Medicine, National Cheng Kung University, Tainan 70403, Taiwan

**Keywords:** podocyte, gelsolin, actin, chronic kidney disease

## Abstract

Chronic kidney disease (CKD) is normally related to proteinuria, a common finding in a compromised glomerular filtration barrier (GFB). GFB is a structure composed of glomerular endothelial cells, the basement membrane, and the podocytes. CKD with podocyte damage may be associated with actin cytoskeleton reorganization, resulting in podocyte effacement. Gelsolin plays a critical role in several diseases, including cardiovascular diseases and cancer. Our current study aimed to determine the connection between gelsolin and podocyte, and thus the mechanism underlying podocyte injury in CKD. Experiments were carried out on Drosophila to demonstrate whether gelsolin had a physiological role in maintaining podocyte. Furthermore, the survival rate of gelsolin-knocked down *Drosophila* larvae was extensively reduced after AgNO_3_ exposure. Secondly, the in vitro podocytes treated with puromycin aminonucleoside (PAN) enhanced the gelsolin protein expression, as well as small GTPase RhoA and Rac1, which also regulated actin dynamic expression incrementally with the PAN concentrations. Thirdly, we further demonstrated in vivo that GSN was highly expressed inside the glomeruli with mitochondrial dysfunction in a CKD mouse model. Our findings suggest that an excess of gelsolin may contribute to podocytes damage in glomeruli.

## 1. Introduction

Chronic kidney disease (CKD) is a significant public health problem on a global scale. Kidney function impairment can result in substantial morbidity [1] and CKD is strongly associated with cardiovascular diseases and is a leading cause of death [2]. CKD patients are more likely to develop peripheral arterial occlusion disease (PAOD). PAOD was found to be significantly associated with low eGFR, low BMI, and high blood pressure in a study of elderly people from the Tianliao area. The relationship between CKD and vascular disease, on the other hand, has not been thoroughly investigated [3]. A healthy human kidney includes one million nephrons. The glomeruli are microvascular units located at the nephron’s beginning that are necessary for plasma filtration and primary urine production. The glomerular filtration barrier (GFB) is made up of fenestrated endothelial cells that line the capillary loops, a specialized glomerular basement membrane (GBM), and podocytes that line the GFB’s exterior [2,4]. The glomerular filtration barrier in the kidney is formed by podocytes, endothelial cells, and the basement membrane [5].

Podocytes play a critical role in maintaining the glomerular filter’s integrity and have been the main focus regarding glomerular research for decades. The interaction of podocytes and endothelial cells in the glomerulus is needed for podocyte dysfunction and depletion in glomerulosclerosis. Endothelial endothelin receptor type A (EDNRA) induces endothelial mitochondrial oxidative stress and endothelial cell dysfunction, which are detrimental variables that result in foot process effacement, podocyte apoptosis, and/or detachment, and ultimately podocyte depletion and glomerulosclerosis [4]. Podocytes are divided into three distinct morphological segments: the cell body, major processes, and foot processes (FPs) [5]. Foot processes must adapt to changing tensile and shear stresses despite lacking contractile actin fibers. The podocyte cell body and major processes contain a network of contractile fibers containing myosin IIA. Tension is generated in the cell body by non-contractile actin filaments in FPs. Focal adhesion complexes that connect integrins to the actin cytoskeleton form and strengthen with localized tension on integrins.

The podocyte foot processes interdigitate with neighbouring podocyte foot processes. The major and minor processes have numerous thread-like nanoprotrusions with bulbous ends into the urinary space. This is called microvillous transformation. The podocyte plasma membrane has depressions of 20–30 nm in size that resemble intramembrane particles. The podocyte filtration barrier is a ladder-like structure with “slit diaphragm” structures overlaying the basal lamina [4]. The filtration slits are connected by a specialized membrane-like cell-to-cell contact called the slit diaphragm. Nephrin and podocin molecules from neighboring foot processes briefly overlap in the slits, forming a dense, zipper-like structure. Following podocyte damage, foot processes become flatter and wider in vivo. This can occur in any glomerular disorder, but it is more common in inflammatory glomerular disorders. When the foot processes retract into primary podocyte processes, the cell body adheres to the GBM [6].

The difficulties inherent in comprehending podocyte biology are exacerbated by those inherent in approaching mammalian nephrons in vivo. Due to the high similarity between nephrocytes and mammalian glomerular podocytes, Drosophila is an appropriate model system for studying podocyte biology in vivo. The primary constituents of the slit diaphragm in Drosophila melanogaster, including nephrin, podocin, and related proteins, are expressed in nephrocytes to filter and detoxify hemolymph [7].

Nephrocytes efficiently pick up secreted fluorescent proteins, indicating that they have no involvement in primary urine generation, but rather function to endocytose hazardous compounds from the hemolymph [7]. The GAL4-UAS system is a highly effective genetic tool for studying Drosophila gene expression and function. Dot-GAL4 is expressed specifically in pericardial nephrocytes [8].

Due to the efficient uptake of secreted fluorescent protein by nephrocytes, they combined the functional readout transgenes MHC-ANF-RFP and the pericardial cell marker Hand-GFP with the nephrocytic-specific driver Dot-GAL4 for targeted gene knockdown, enabling the identification of genes involved in nephrocytes function [9].

Within a cell, changes in the actin cytoskeleton are required for the maintenance of cell shape, motility, and intracellular transport. Actin filaments are arranged in a variety of ways to perform these functions [6]. Gelsolin is a calcium-dependent, multifunctional actin-regulatory protein that engages in actin filament severing and capping as well as actin remodeling. The process of weakening the bonds between actin molecules within a filament, resulting in the filament splitting in half, is known as Severing actin filaments... Following severance, gelsolin remains attached to the barbed (or “plus”) end of the filament and forms a cap, resulting in the formation of short actin filaments that cannot reanneal or elongate at their barbed ends. This mechanism allows Gelsolin to disassemble actin networks. By binding to actin monomers, gelsolin can also act as a nuclease [10].

Puromycin aminonucleoside (PAN) treatment harmed the podocyte cytoskeleton, retracted the FP, and induced pathological remodelling; the cytoskeletal network vanished and cell integrity was compromised [11]. Additionally, PAN increases the expression of a Ca2+ channel known as the Transient Receptor Potential Cation Channel 6 (TRPC6), which has been observed in proteinuria kidney disease [12]. The purpose of this study is to ascertain how gelsolin regulates cytoskeleton assembly, specifically actin assembly, in podocytes during chronic kidney injury processes.

## 2. Results

### 2.1. Gelsolin Is Required for Maintaining Nephrocyte Function in Drosophila

**Figure 1:**
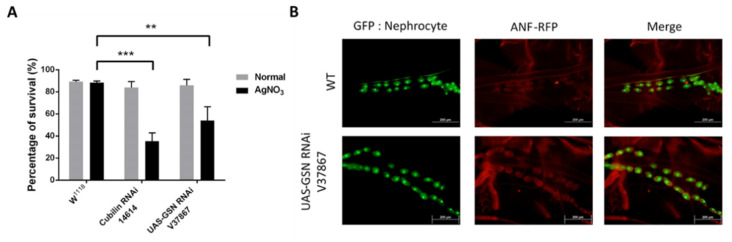
(**A**) Survival of gelsolin-knocked down *Drosophila* after AgNO_3_ exposure. The W1118 strain of Drosophila was used to represent wild-type Drosophila, and Cubilin knockdown flies served as a positive control in this toxin assay. Although gelsolin-knockdown Drosophila did not show altered viability under normal conditions, their rate of survival was dramatically reduced under stress conditions caused by AgNO3 toxin. *n* = 300, ** *p* < 0.01, *** *p* < 0.001. (**B**). Dissection of Drosophila nephrocytes. Nephrocytes from gelsolin-deficient Drosophila accumulate more ANF-RFP. (ANF = Atrial natriuretic factor).

Drosophila nephrocytes act as hemolymph filters. Due to the fact that nephrocytes are capable of removing toxins such as silver nitrate (AgNO3) from hemolymph [7], we investigated whether gelsolin is required for nephrocyte toxin removal function to be maintained. Cubilin-mediated protein reabsorption has been reported to be required for toxin removal [13] and we thus used it as a positive control in our AgNO3 toxin assay. We discovered that, despite the gelsolin-knocked down while Drosophila remained viable under normal conditions, the survival rate of flies was significantly reduced when stressed with AgNO3 (** *p* < 0.01; Figure 1A). Combining a Hand-GFP, MHC-ANF-RFP transgenic strain expressing secreted fluorescent proteins with a nephrocyte-specific driver with a gelsolin knockdown strain, we can study the genes required for nephrocyte function using Drosophila. The results indicated that gelsolin-deficient Drosophila nephrocytes accumulated more ANF-RFP (Figure 1B). These findings indicated that gelsolin is required for nephrocyte function, which is to remove toxins from the hemolymph, and thus for animal survival. 

### 2.2. Podocyte Cell-Height and Stiffness were Altered by PAN Exposure

**Figure 2:**
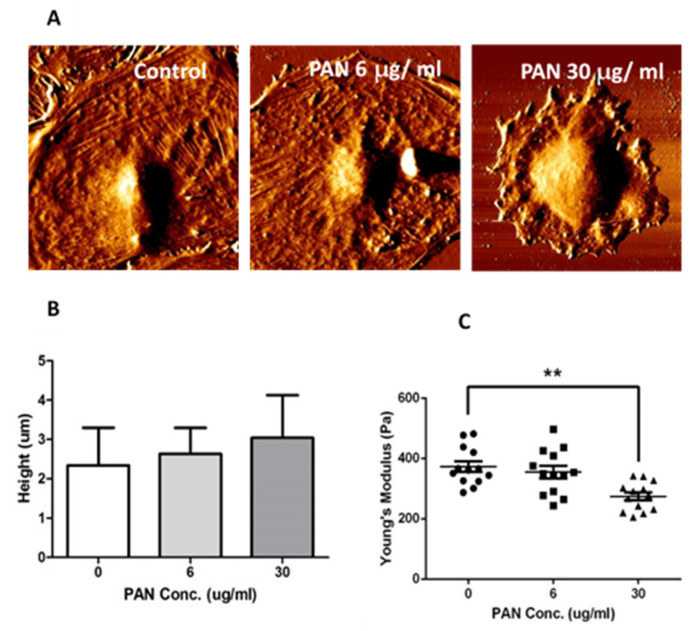
Atom force microscopy (AFM) images of podocytes. (**A**) The actin cytoskeleton network was disrupted after PAN exposure. (PAN = Puromycin aminonucleoside). (**B**) Podocyte height increased slightly with increasing PAN concentrations. (PAN = Puromycin aminonucleoside). (**C**) Podocyte stiffness decreased significantly with increasing PAN concentrations. (PAN = Puromycin aminonucleoside). ** *p* < 0.01.

**Figure 3:**
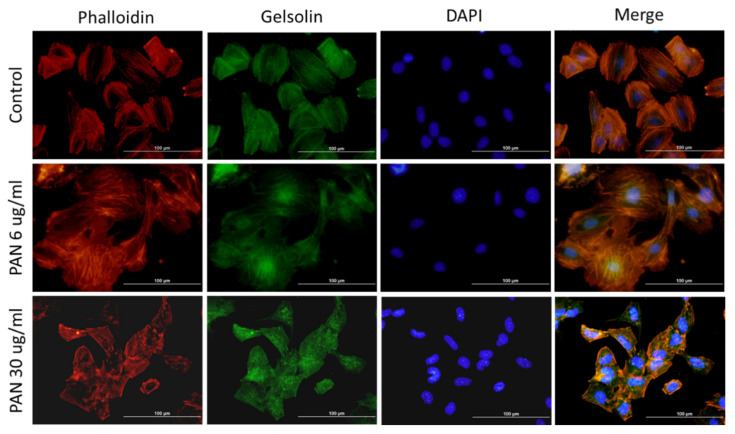
Immunofluorescence staining images of podocytes. Phalloidin staining showed that PAN exposure-induced actin cytoskeleton rearrangement in podocytes and gelsolin proteins co-localize with actin filaments. (PAN = Puromycin aminonucleoside).

To determine whether PAN exposure disrupts the actin cytoskeleton of podocytes and thus results in morphological changes in podocytes, we scanned podocyte surfaces and measured cell height and stiffness using AFM. AFM is a type of scanning-probe microscopy that creates images of samples by scanning them with a physical probe [14]. The podocyte actin cytoskeleton network is clearly visible in the control group, and podocytes appear to be flatter on the coating dish than those exposed to PAN. As PAN concentrations increased, the actin cytoskeleton network became more distorted and cell size decreased (Figure 2A). Although the height of podocyte cells did not differ significantly between PAN concentrations, it did show a positive trend with increasing PAN concentration (Figure 2B). The stiffness of podocyte cells is comparable to its own elastic modulus and is denoted by Young’s modulus [14]. The data showed that podocyte cell stiffness significantly decreased with increasing PAN concentrations (** *p* < 0.01; Figure 2C). These findings suggested that PAN exposure disrupted the podocyte actin cytoskeleton network, resulting in changes in cell morphology and stiffness. We used immunofluorescence staining to confirm our findings regarding the state of the actin cytoskeleton network following PAN treatment. To visualize actin filaments, phalloidin was used (Figure 3). After PAN treatment, the actin cytoskeleton network fragmented, but not in the control group. Gelsolin and actin were clearly co-localized upon double staining with gelsolin and phalloidin.

### 2.3. Gelsolin Expression Increased in PAN-Damaged Podocytes

**Figure 4:**
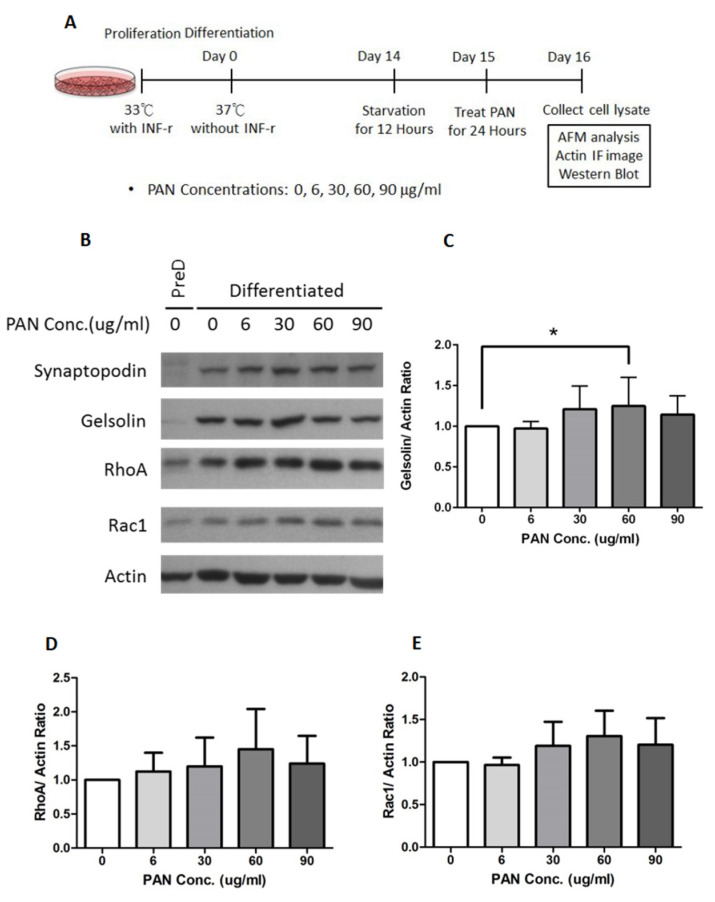
Protein expression by PAN-treated podocytes examined by Western Blot. (**A**). Podocyte cells were cultivated at 33 °C with 5% CO_2_ to allow proliferation, and then at 37 °C for 14 days to induce differentiation. After starvation for 12 h, podocytes were treated with different concentrations of PAN for 24 h, and subsequently analyzed. (PAN = Puromycin aminonucleoside). (**B**). Expression of the proteins synaptopodin, gelsolin, RhoA, Rac1, and actin after PAN treatment were demonstrated by Western Blot. (**C**). Relationship between gelsolin: actin ratio and PAN concentration. Histograms represent mean ± SEM. Data were analyzed by one-way ANOVA. (**D**). Relationship between RhoA: actin ratio and PAN concentration. Histograms represent mean ± SEM. Data were analyzed by one-way ANOVA. (**E**). Relationship between Rac1: actin ratio and PAN concentration. Histograms represent mean ± SEM. Data were analyzed by one-way ANOVA. * p < 0.05.

To investigate how gelsolin affects actin assembly in podocytes during the CKD process, we used cultured podocyte cells and allowed to differentiate for 14 days at 37 °C. After 12 h of starvation, podocyte cells were switched to FBS-free 1640 RPMI medium. Following starvation, cells were treated for 24 h with one of five different concentrations of PAN (0, 6, 30, 60, or 90 µg/mL). Cells were then collected, proteins were extracted using Western blot (Figure 4A,B), and protein expression was analyzed.

Synaptopodin is a critical marker for differentiated podocytes [15]. It was used to ensure that the podocyte cells had fully differentiated prior to the experiments (Figure 4B). Gelsolin levels increased significantly as PAN concentration increased (* *p* < 0.05; Figure 4C). RhoA and Rac1 are Rho GTPases that regulate a variety of cell behaviors, including actin dynamics [16,17]. Additionally, the expression of RhoA and Rac1 increased in proportion to the PAN concentration (Figure 4D,E).

### 2.4. PAN-Induced CKD Mice Exhibited Effacement of the Podocyte Foot Process

**Figure 5:**
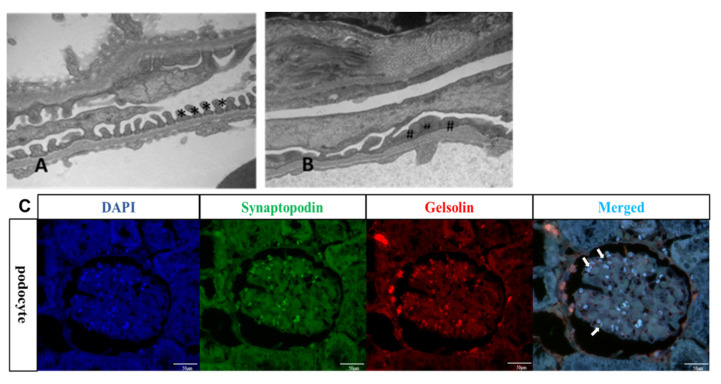
TEM images of mouse glomerular filtration barrier and IF images of mouse podocytes. (**A**) Podocytes of control mice (Saline injection) appear intact (*) under TEM. (Magnification: 30,000×). (**B**) Podocytes of PAN mice showed effacement (#) under TEM. (Magnification: 30,000×). (**C**) Representative micrographs showing immunostained of synaptopodin (green) and gelsolin (red) on glomerular. Podocytes were shown in a white arrow. (Magnification: 400×). (PAN = Puromycin aminonucleoside).

Renal biopsies of patients with proteinuria frequently reveal effacement of the podocyte foot process [18]. Generated this condition C57BL/6 mice were intravenously injected with 450 mg/kg PAN to induce podocyte failure. The glomeruli of PAN mice were analyzed using transmission electron microscopy. The three layers of the filtration barrier as well as the intact podocyte structure are visible in Figure 5A (indicated by asterisks *). In comparison, PAN mice podocytes exhibited severe effacement (Figure 5B, denoted by # symbols). IF images also show that PAN mice podocytes highly expressed gelsolin (Figure 5C).

### 2.5. Gelsolin was Abundantly Expressed in the Glomeruli of Chronic Kidney Disease (CKD) Mice Models

**Figure 6:**
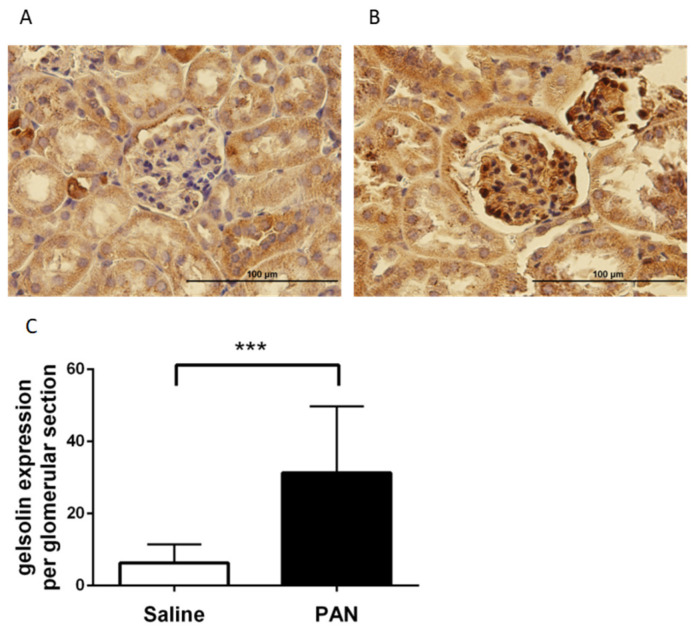
Histological analysis of gelsolin expression in the glomeruli of PAN mice. (**A**) IHC staining of gelsolin expression in kidney sections of control mice. (Magnification: 400×). (**B**) IHC staining of gelsolin expression in kidney sections of PAN mice. (Magnification: 400×) (PAN = Puromycin aminonucleoside). (**C**) Quantitative comparison of gelsolin induction after injection. *** *p* < 0.001.

**Figure 7:**
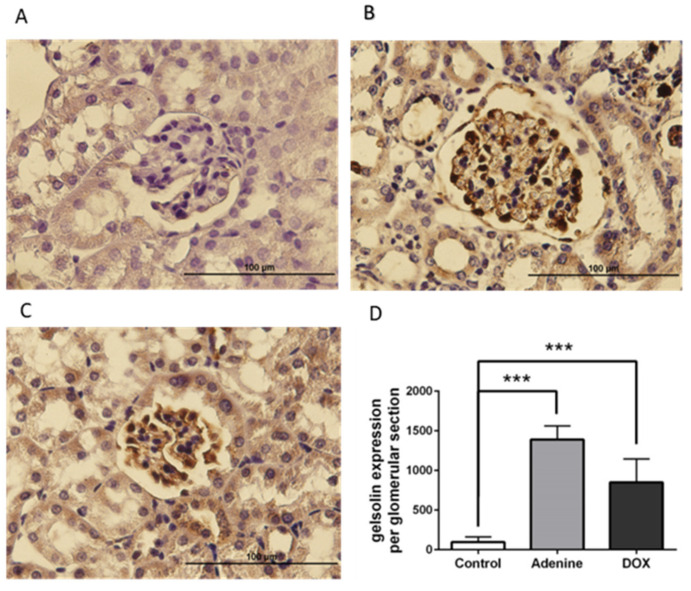
Histological analysis of gelsolin expression in mouse glomeruli. (**A**) Immunohistochemistry staining of gelsolin expression in kidney sections of control mice. (Magnification: 400×). (**B**) Immunohistochemistry staining of gelsolin expression in kidney sections of mice injected with 0.25% adenine. (Magnification: 400×). (**C**) Immunohistochemistry staining of gelsolin expression in kidney sections of mice injected with doxorubicin. (Magnification: 400×). (**D**) Quantitative comparison of gelsolin induction after saline, adenine, and doxorubicin injection. *** *p* < 0.001.

Gelsolin expression was determined histologically in the kidneys of mice injected with saline or PAN. Gelsolin induction was observed in the glomeruli of PAN mice following IHC staining (Figure 6B), but not in the glomeruli of control mice injected with saline (Figure 6A). These findings indicate that gelsolin accumulates in the glomeruli of PAN mice, which contain podocytes. Additionally, we examined the glomeruli of animal models of renal failure induced by other chemicals. Gelsolin expression was also significantly increased in the glomeruli of the control group (Figure 7A) when 0.25% adenine (Figure 7B) or doxorubicin was injected (Figure 7C).

## 3. Discussion

In this study, we evaluated whether elevated gelsolin levels in patients with chronic kidney disease may be an indication of podocyte damage. As previous research conducted that there is relation between CKD, low eGFR, and vascular diseases, we focused on gelsolin as Gelsolin-knocked down Drosophila exposed to AgNO3 had a significant reduction in survival, indicating that gelsolin played a physiological role in the maintenance of nephrocyte function. PAN treatment disrupted the actin cytoskeleton network in podocyte cell culture. The levels of actin-binding protein, gelsolin, increased as PAN concentrations increased. Gelsolin levels also significantly increased in mice treated with adenine and doxorubicin compared to controls. Mice which injected with higher concentration of PAN exhibited albuminuria and podocyte effacement. Gelsolin exists in three isoforms, two of which are found in the cytoplasm and one of which is found in the secreted extracellular plasma. Each isoform is encoded by a single gene [10,19]. The secreted gelsolin is distinguished from its cytoplasmic isoform by an additional N-terminal peptide [19]. Plasma gelsolin levels have been detected in a variety of disease states and animal models. Plasma gelsolin is an extracellular actin scavenger system (EASS) component that is capable of severing circulating actin filaments [19,20]. Cell membrane disruption, followed by cell death, results in the release of intracellular proteins into the systemic circulation. Actin filaments released by dying cells into the bloodstream can cause damage to microvascular capillaries and active plates, as well as increasing clot propagation, which can be fatal [21,22]. EASS is a plasma-based actin severing and clearance system that protects against the potentially harmful effects of circulating actin. Plasma gelsolin levels have been shown to be a significant prognostic biomarker for human health and disease conditions. They are significantly decreased in several diseases and inflammatory conditions, including severe trauma, cardiovascular diseases, and hemodialysis [23,24]. Recent study showed that hemodialysis decreased the mortality rate of patients with ESRD (end-stage renal disease). After initiating hemodialysis, non-survivors had significantly lower mean plasma gelsolin levels than survivors [24]. Plasma gelsolin concentration is related to eGFR, i.e., plasma gelsolin levels are associated with impaired renal function. Due to the dynamic nature of the actin cytoskeleton within cells, it is difficult to ascertain the precise state of gelsolin effects on actin. However, since gelsolin is a calcium-regulated actin severing and capping protein, determining the cell calcium concentration using the Fura-2 fluorescent technique may assist in answering the question. Furthermore, combining with the G-actin/F-actin assay, this can aid in elucidating actin’s gelsolin function. Gelsolin is now being demonstrated to be a multifunctional regulator of cellular signalling, apoptosis, and cancer epithelial mesenchymal transition via mechanisms distinct from those that regulate actin [19,25]. Gelsolin acts as both an inducer and a suppressor of apoptosis. Certain types of renal injury may result in podocyte apoptosis, and thus apoptosis is another intriguing issue associated with gelsolin and podocyte injury. Gelsolin overexpression in nephrocytes is a lethal condition in the Drosophila model. Since the nephrocyte’s primary function is to filter the hemolymph, the overexpression of gelsolin in Drosophila disrupted nephrocyte function, resulting in toxin accumulation and thus death. However, the mechanism by which gelsolin overexpression results in lethality is unknown. Although the absence of gelsolin had no effect on Drosophila viability under normal conditions, the survival rate of these flies was significantly reduced when exposed to AgNO3 toxin. Gelsolin is required for the removal of toxins from hemolymph, which is critical for nephrocytes’ survival. In our in vivo data, we used PAN to induce podocyte injury. We used PAN to induce podocyte injury in an animal model. Shimo et al. successfully generated nephrosis mice model by intraperitoneally administering 450 mg/kg PAN to C57BL/6 mice [26]. This method did not work for us, however. Therefore, we switched to intravenous injection with the same dose for the generation of PAN mice, but the vast majority of mice (70%) died immediately following the injection. Hence, the optimal PAN concentrations for the generation of a mice model of podocyte injury remain to be optimized. 

## 4. Materials and Methods

### 4.1. Fly Strains

We examined the following Drosophila strains, (1) W1118, (2) USA-GELSOLIN RNAi (VDRC stock 37,867), and (3) UAS-CUBILIN RNAi, which were obtained from the Vienna Drosophila RNAi Center and the Bloomington Drosophila stock center. In addition, MHC-ANF-RFP, Hand-GFP, Dot-Gal4 strain was obtained from Professor Zhe Han’s lab in the Children’s National Health System in Washington, DC, USA. The strain MHC-ANF-RFP, Hand-GFP, Dot-Gal4 crossed with W1118 was used as wild-type files. Flies were reared on standard food at 25 °C. Interventional studies involving animals or humans, and other studies that require ethical approval, must list the authority that provided approval and the corresponding ethical approval code.

### 4.2. AgNO3 Toxin Assay for Nephrocyte Function

*Drosophila* with appropriate genotypes were crossed and allowed to lay eggs on apple juice agar plates for 24 h. After 24 h, the newly hatched first-instar larvae on the plates were transferred to normal standard food tubes with or without AgNO_3_ (0.005%). Flies were incubated at 25 °C to allow development until adulthood, and fly survival (%) was scored.

### 4.3. Nephrocyte Dissection

Third-instar larval nephrocytes were dissected in phosphate-buffered saline (PBS). They were fixed in 4% paraformaldehyde for ten minutes and then washed four times with PBS. Nephrocytes were mounted on glass slides following washing, and images were captured using a fluorescence microscope (BX51, Olympus system microscope).

### 4.4. Culture of Podocyte Cells

Professor Yu-Hsiang Hsu from National Cheng Kung University generously provided immortalized podocytes from mice. Podocyte cells were maintained in RPMI 1640 medium supplemented with 10% FBS and 1% penicillin/streptomycin (AMIMED^®^ by BioConceptTM). To propagate podocytes, cells were grown at 33 °C with 5% CO_2_ in dishes coated with type I collagen (permissive conditions) in culture medium supplemented with 10 U/mL recombinant interferon- (IFN-). To induce differentiation, podocytes were maintained in culture medium for at least 14 days without IFN- (non-permissive conditions) at 37 °C with 5% CO_2_.

### 4.5. Bicinchoninic Acid (BCA) Protein Assay for Quantification of Protein Concentration

We diluted cell lysate samples ten-fold with Milli-Q water and vortexed to mix in Eppendorf tubes. Hence, 25 L of standard and diluted protein samples were pipetted into 96 well plates. We added 200 L working solution to the standards and diluted samples in the well plates. The working solution contains 50 parts BCA solution and one part copper solution G-Biosciences (St. Louis, MO, USA). Under light exclusion, we incubated the well plates at 37 °C for 30 min. We determined protein concentrations by measuring light absorbance at 562 nm using a SpectraMaX 340PC384 Molecular Device and constructing a standard curve.

### 4.6. Western Blot Analysis of Gelsolin’s Effect on Podocytes

To determine the effects of gelsolin on podocytes, and specifically the role of gelsolin in PAN-induced podocyte injury, 1*105/10 cm dish podocyte cells were seeded and allowed to differentiate for 14 days. Prior to application of the drug, podocyte cells were starved for 12 h in FBS-free RPMI 1640 medium. Following this starvation interval, cells were treated with PAN in FBS-free medium at concentrations of 0, 6, 30, 60, or 90 µg/mL. After 24 h of PAN treatment, podocyte cell lysates were collected in RIPA buffer containing a protease inhibitor cocktail and protein concentrations were determined using a BCA protein assay. Protein samples were then prepared with 6X loading dye and heated to 100 °C for ten minutes based on the BCA assay result. Electrophoresis of protein samples in 10% polyacrylamide gel was performed at 50 V for 30 min and at 100 V for approximately two hours. Proteins were then transferred to PVDF membranes for three hours at a current of 300 mA. After transferring processes, membranes were blocked in 5% skim milk at room temperature for one hour and then washed three times for ten minutes each with TBST (Tris-buffered saline solution containing 1% Tween-20 detergent). Ten transfer membranes were incubated overnight at 4 °C with primary antibodies (1:1000 in TBST). Synaptopodin, gelsolin, RhoA, Rac1, and actin were used as primary antibodies in this study. The following day, transfer membranes were washed three times with TBST buffer and then immersed for one hour at room temperature in secondary antibodies (goat anti-mouse or anti-rabbit IgG at 1:10,000 in 5% skim milk). Transfer membranes were washed twice with TBST and then incubated in ECL reagents before being exposed to X-ray film in the darkroom.

### 4.7. Phalloidin Actin Staining

To visualize the actin cytoskeleton and localize gelsolin in podocyte injury induced by PAN, 1*104/6-well plate podocyte cells were seeded onto coverslips and allowed to differentiate for 14 days. After 12 h of starvation, cells were treated for 24 h with PAN at concentrations of 0, 6, 30, 60, or 90 µg/mL in FBS-free medium. Following PAN exposure, we washed the coverslips twice with PBS and fixed the cells for 30 min at room temperature in 4% paraformaldehyde. Following that, we removed the paraformaldehyde and neutralized it for 30 min at room temperature with 0.1 M glycine. For perforation, we immersed coverslips in 0.5 percent Triton-X-100 for 30 min and then blocked them for one hour with 3 percent bovine serum albumin in PBS. The following steps were carried out in the absence of light. Gelsolin antibodies were diluted in PBS (1:1000) with 1% bovine serum albumin and pipetted onto glass slides. Following two hours of washing with PBS three times, we added the second antibody (goat anti rabbit conjugated FITC) for one hour. Gelsolin staining was performed after three PBS washes. For 30 min, we incubated the coverslips with phalloidin conjugated with CF-543 (Biotium, Fremont, CA, USA). We stained the nuclei with DAPI for ten minutes after three PBS washes, mounted the coverslips on the slides with mounting medium, and allowed them to air-dry. A fluorescence microscope was used to conduct the observations and photography (BX51, Olympus system microscope).

### 4.8. Determination of Podocyte Cell Height and Stiffness by Atom Force Microscopy (AFM)

To visually assess whether PAN treatment disrupted the actin cytoskeleton of podocytes and altered their stiffness and morphology, 1*104/6 cm dish podocyte cells were seeded and allowed to differentiate for 14 days. After 12 h of starvation, cells were treated for 24 h with PAN at concentrations of 0, 6, 30, 60, or 90 µg/mL in FBS-free medium. Podocyte cells were washed with PBS following PAN exposure. Atomic force microscopy was used to determine the height and stiffness of podocytes. Standard silicon-nitride cantilevers with a spring constant of 0.03 N/m were used in this study. All measurements were conducted at room temperature in PBS, and the data were analyzed using the JPKSPM data processing software.

### 4.9. PAN-Induced Effacement of the Podocyte Foot Process

Wild-type C57BL/6 mice (*n* = 6, 8–10 weeks of age, weight 20–25 g) were obtained and divided into two groups at the National Cheng Kung University Laboratory Animal Center. One group received 450 mg/kg PAN intravenously, while the other group (control group) received normal saline solution intravenously. Every two days, body weight was determined. The mice were sacrificed ten days after PAN or saline injection and their kidneys were rapidly removed.

### 4.10. Immunohistochemistry Staining of Kidney Sections

Mice kidneys were fixed in 4% paraformaldehyde and paraffin embedded. Perfusion, dehydration, clearance, infiltration, and paraffin embedding of kidney tissues were followed by sectioning the tissues into 5-m thick sections and transferring them to slides. After 15 min at 65 °C, samples were immersed in xylene and ethanol at increasing concentrations of 100%, 95%, and 75% to dissolve the paraffin. We retrieved samples at 100 °C for 30 min using sodium citrate buffer (10 mM, pH6.0). After samples were cooled to room temperature, slides were washed three times with 1% PBST on the shaker for five minutes each. After ten minutes of light exclusion, the slides were immersed in 3 percent H_2_O_2_ at room temperature. After three PBST washes, we blocked for one hour at room temperature in 1% BSA buffer. Primary antibodies were applied to slides at room temperature for one hour and then stored at 4 °C overnight. The following day, slides were incubated for one hour at room temperature with the second antibody (1:00). We stained the nuclei with hematoxylin and DAB chromogen substrate stain (Dako, Agilent Technologies, Denmark). Photographs of the slides were taken using an Olympus BX51 system microscope.

### 4.11. Transmission Electron Microscopy (TEM) of Kidney Sections

Zoletil (Virbac, France) was used to anaesthetize mice, and their kidneys were rapidly dissected, cut into 0.5 cm-thick sections, and immersed in 2.5% glutaraldehyde (diluted in 0.1 M cacodylate buffer, pH 7.2). After two hours of fixation, kidney sections were washed three times for 15 min at 4 °C in 0.1 M cacodylate buffer with 5% sucrose. After several rinses, kidney samples were post-fixed in 1% osmium tetroxide for one hour at 4 °C in the dark, followed by two 15-min rinses in 0.1 M cacodylate buffer with 5% sucrose. Kidney samples were dehydrated in a graded ethanol series (50%, 70%, 80%, 90%, and 100% ethanol) for 15 min each and then transferred to propylene oxide for 30 min prior to six hours of embedding in Embed-812. Then, 0.8-m-thick sections were cut using a rotary microtome and stained with 1% toluidine blue. The kidneys were cut into ultra-thin 90 nm sections and mounted on slot grids. Under light exclusion, sections were stained with 2% uranyl acetate in 50% ethanol for 20 min and then contrasted with lead citrate for three minutes. The sections were viewed and photographed using a Hitachi H-7650 transmission electron microscope equipped with a CCD camera.

### 4.12. Statistical Analysis

All results are expressed as mean ± standard error of the mean (SEM). The data obtained from two independent experiments were analyzed with Student’s *t*-test (two tailed), with a significance level of *p* < 0.05 (* = *p* < 0.05; ** = *p* < 0.01; *** = *p* < 0.001).

## 5. Conclusions

Enhanced gelsolin levels in chronic kidney disease patients may reflect damaged podocytes and advanced stages of CKD. Gelsolin may play a pathophysiological role in the in vitro and in vivo animal model of PAN-induced podocyte failure. A limitation of data was that gelsolin levels were measured after induction of podocyte damage in three different animal models, namely injection of PAN, adenine and doxorubicin. These models induce different levels of podocyte injury and therefore variable increases in gelsolin levels.

## Data Availability

The data presented in this study, as well as supporting data, are available upon request from the corresponding authors.
